# Prenatal oestrogen-testosterone balance as a risk factor of migraine in adults

**DOI:** 10.1186/s10194-021-01326-3

**Published:** 2021-10-07

**Authors:** Magdalena Kobus, Aneta Sitek, Bogusław Antoszewski, Jacek Rożniecki, Jacek Pełka, Elżbieta Żądzińska

**Affiliations:** 1 Department of Anthropology, Faculty of Biology and Environmental Protection, University of Lodz, 90-237 Lodz, Poland; 2 Department of Plastic, Reconstructive and Aesthetic Surgery, Institute of Surgery, Medical University of Lodz, Lodz, Poland; 3 Department of Neurology, Stroke and Neurorehabilitation, Medical University of Lodz, Lodz, Poland; 4 Department of Neurology, Norbert Barlicki Memory University Teaching Hospital, Lodz, Poland; 5 Biological Anthropology and Comparative Anatomy Research Unit, School of Medicine, The University of Adelaide, Adelaide, South Australia 5005 Australia

**Keywords:** Migraine, Sex hormones, Sex steroids, Digit ratio, 2D:4D, Prenatal, Oestrogen, Testosterone, Intrauterine development

## Abstract

**Background:**

Migraine is a common neurological disease with extremely debilitating, but fully reversible symptoms. Women suffer from migraine more often than men. It was assumed that fluctuation of oestrogen level during menstrual cycle is one of many factors responsible for more frequent migraine attacks. The second-to-fourth digit ratio (2D:4D) is considered as an indicator of prenatal sex steroids. Balance of prenatal androgens (testosterone) and oestrogen has been studied in numerous diseases that are affected by hormones. However, the relationship between migraine and the sex steroids balance in prenatal development is still unexplained.

The aim of this paper is to provide an evidence of relationship between prenatal oestrogen and testosterone exposure following 2D:4D digit ratio, and migraine prevalence in adults.

**Methods:**

We examined a group of 151 adults (33 males, 118 females) with migraine and a control group of 111 adults (45 males, 66 females). 2D:4D digit ratio of both hands was measured using sliding Vernier calliper.

**Results:**

Significant differences were found in the right hand. Female migraineurs had lower value of 2D:4D ratio than the control group and the right 2D:4D was lower than left 2D:4D (Δ2D:4D), suggesting prenatal testosterone dominance. The opposite relationship was observed in males. Male migraineurs had higher value of 2D:4D ratio and Δ2D:4D was greater than the control group, suggesting prenatal oestrogen dominance.

**Conclusions:**

Our results suggest that depending on sex, different proportion of prenatal sex steroids might be a risk factor of migraine in adults. Women with migraine were presumably exposed in prenatal life to higher testosterone levels relative to oestrogen, while men with migraine were probably exposed in prenatal life to higher levels of oestrogen relative to testosterone.

## Background

Migraine is one of the most common primary headaches next to tension-type headache. International Headache Society in Headache classification (ICHD-3) distinguishes several types of migraine. Two major types are migraine without and with aura. Aura symptoms are fully reversible and they can be visual, sensory, speech, motor, brainstem and retinal [1]. Numerous epidemiological studies report widespread prevalence of migraine and its burdensome health effects and socio-economic costs [2–4].

Migraine attacks affect over 11% of the world’s population and occur 3 times more often in women than in men [5–7]. It should be underlined that also about 10% of children suffer for migraine. It means that in some cases migraine influences quality of life already in childhood [4, 8]. Nevertheless, recent studies have shown that migraine is still underdiagnosed and undertreated [2, 9].

Research about pathophysiological mechanisms of migraine evolve continuously due to the fact that migraine is probably a multifactorial disease [10–14]. Migraine is definitely dependent on genetic predisposition [15–17], while attacks can be triggered by various endogenous and environmental factors [18–21]. Prenatal factors of migraine have not been widely studied so far [22–24]. The authors of this paper aimed to analyse the influence of prenatal sex steroid exposure (2D:4D ratio) as a potential risk factor of migraine.

The ratio of 2nd and 4th digit length (2D:4D) in human hand is a well-known method to assess sex steroids proportions during prenatal life. Males usually have lower ratio (due to longer 4th digit) than females (due to shorter 4th digit) [25]. The research with mice conducted by Zheng and Cohn confirmed hypothesis that activity of androgen and oestrogen receptors in prenatal life has influence of a digit growth. Inactivation of androgen receptor decreased growth of 4th digit (led to a feminized 2D:4D) while inactivation of oestrogen receptor α increased growth of 4th digit (led to a masculinized 2D:4D). 4D has higher levels of both receptors and their activity influences 2D:4D digit ratio by regulating expression of 19 skeletogenic genes [25]. Difference between 2D:4D R and L (Δ2D:4D) is also sexually dimorphic and tends to be negative in males and positive in females [26]. Low Δ2D:4D is thought to be another correlate of high prenatal testosterone levels and low prenatal oestrogen levels [27]. Δ2D:4D was first considered to be a correlate of prenatal sex steroids by Manning et al. [28] and further by Manning & Peters in studies concerned with handedness [29]. The 2D:4D digit ratio seems to be the risk factor of many diseases in different populations, among others with breast cancer [30], coronary heart disease [31], ischemic stroke [32] and lung cancer [33].

Geschwind and Galaburda (1985) were the first who hypothesised that prenatal testosterone slows down the growth of certain areas of the left hemisphere and promotes the growth of the homologous areas of the right hemisphere. Researchers linked high prenatal testosterone levels with aetiology of e.g. autism and migraine [34]. The issue whether 2D:4D levels are associated with autism is still under investigation [35–41]. Until now the problem of the relationship between digit ratio and primary headaches has been addressed only in 1 original publication. Xie et al. showed in Chinese population that females with migraine or with tension-type headache had lower 2D:4D ratio than females in the control group without these primary headaches [22].

The main aim of this study was to evaluate if proportion of sex steroids exposure during pregnancy is a possible risk factor of migraine in Polish population.

## Material and methods

We recruited patients of Norbert Barlicki Memory University Teaching Hospital No. 1 in Lodz. The study included two groups of adults. Migraineurs were recruited from the outpatient neurological clinic of the hospital (151 participants aged 18–76 years), while patients without migraine and other headaches declared were recruited from other hospital clinics (111 participants aged 21–74 years). Data collection started in 2019 and ended in 2020.

Measurements of the length of the 2nd and 4th digits were performed by a professional staff from the Department of Anthropology of the University of Lodz. Sliding Vernier calliper was used in accordance with standard anthropometric procedure of Martin measurements [42]. The measurements of digit lengths in both hands was made between *pseudophalangion* and *dactylion* points [43]. Direct finger measurement is accurate method to determine 2D:4D R [44, 45].

Based on these measurements the 2D:4D digit ratios and the difference of 2D:4D ratio between the hands (Δ2D:4D = 2D:4D R - 2D:4D L) were calculated.

Statistical analysis of the results was performed in the STATISTICA 13.0 program. All statistical calculations were carried out in spreadsheets after anonymization of the data. Age distribution, 2D:4D R & L digit ratios and Δ2D:4D normality were assessed using the Shapiro-Wilk test. Age distribution was skewed - Mann-Whitney test (Z) was used to assess intergroup and sex differences of this trait. 2D:4D R, 2D:4D L and Δ2D:4D had normal distributions. Relationship 2D:4D R & L with age and Δ2D:4D with age were accessed by Spearman’s rank correlation coefficient (R). Mean values of 2D:4D R & L and Δ2D:4D in women and men as well as in the migraine group and the control group were compared using contrast analysis. Due to the fact that during contrast analysis multiple comparisons were made probability of making a type I error could increase. On the other hand applying stringent multiple testing corrections (Bonferroni correction; Holm’s Sequential Bonferroni Procedure) may increase the probability of making a type II error. In order to make a reasonable decision regarding significance of testing differences probability of obtaining a certain number of statistically significant tests among the total number of performed multiple comparisons was calculated according to the following formula:

$$ {\mathrm{P}}_{\mathrm{B}}\left(\mathrm{A}\right)={\alpha}^{\mathrm{A}}{\left(1-\alpha \right)}^{\mathrm{B}-\mathrm{A}} $$

where: A- number of statistically significant results, B- total number of tests performed, α- significance level [46]. Odds ratios (ORs) were estimated with 95% confidence intervals (CIs). Cohen’s effect sizes (Cohen’s Δ) were calculated using Online Effect Size Calculator (https://www.socscistatistics.com/effectsize/default3.aspx).

## Results

Median age for females with migraine was 41,5 years (interquartile range from 29 to 51) while for females in the control group - 43 years (IQR 31–51). Half of males in the migraine group were under 36 (IQR 27–50) and half of males in the control group were under 42 (IQR 31–46). No statistically significant differences were found in age between sexes and the both study groups (F/M) also (Table 1).

**Table 1:** Statistical age characteristics for the study group and the control group (F/M)

Characteristic	Migraine group	Control group	Migraine group vs Control group
Females			
n	118	66	Z = -0.52; *p* = 0.604
Median	41.5	43.0	
Q_25_- Q_75_	29–51	31–51	
Males			
n	33	45	Z = -0.55; *p* = 0.581
Median	36.0	42.0	
Q_25_- Q_75_	27–50	31–46	
Females vs Males	Z = 0.82; *p* = 0.410	Z = 0.77; *p* = 0.440	

n-sample size, Q_25_- Q_75_ - interquartile range; Z-values Manna-Whitney for *n* > 20, p- probability

There was no correlation between age and 2D:4D R & L and Δ2D:4D in the both female and the male. No statistically significant correlation was also found between age and 2D:4D R & L and Δ2D:4D in the female and the male separately in both study groups (Table 2).

**Table 2:** Correlation between age and 2D:4D digit ratios

Groups / Sex		n	2D:4D R & age		2D:4D L & age		Δ2D:4D & age	
			R	p	R	P	R	p
Females		184	−0.08	0.290	− 0.06	0.418	−0.03	0.650
Males		78	− 0.01	0.977	0.06	0.574	0.04	0.703
Migraine group	**F**	118	−0.05	0.596	− 0.003	0.976	0.002	0.981
	**M**	33	0.05	0.780	−0.06	0.747	0.12	0.501
Control group	**F**	66	−0.20	0.105	−0.12	0.321	−0.11	0.374
	**M**	45	−0.07	0.655	−0.15	0.320	0.23	0.128

n-sample size, R- Spearman’s rank correlation coefficient, p- probability

Table 3 presents average values and standard deviations of 2D:4D R & L and Δ2D:4D in the both study groups (F/M). Table 4 presents Cohen’s effect sizes calculated to assess the magnitude of the mean differences and most of them was medium.

**Table 3:** Statistical characteristics of 2D:4D in migraine and control groups

Characteristic	Migraine group			Control group		
	n	x	SD	n	x	SD
Females						
2D:4D R	118	0,9869	0,0292	66	10,056	0,0297
2D:4D L		0,9958	0,0293		0,9969	0,0304
Δ2D:4D		−0,0089	0,0324		0,0087	0,0299
Males						
2D:4D R	33	0,9907	0,0335	45	0,9726	0,0233
2D:4D L		0,9864	0,0222		0,9855	0,0244
Δ2D:4D		0,0043	0,0348		−0,0129	0,0189

**Table 4:** Contrast analysis results

Characteristic	Females vs Males						Migraine group vs Control Group					
	Migraine group		Cohen’s Δ	Control Group		Cohen’s Δ	Females		Cohen’s Δ	Males		Cohen’s Δ
	F	p		F	p		F	p		F	p	
2D:4D R	0.46	0.500	0.12	**34.55**	**< 0.001**	1.24	**17.64**	**< 0.001**	0.64	**7.41**	**0.007**	0.63
2D:4D L	2.88	0.091	0.36	**4.42**	**0.036**	0.41	0.07	0.793	0.04	0.02	0.889	0.04
Δ2D:4D	**4.94**	**0.027**	0.39	**13.63**	**< 0.001**	0.86	**14.35**	**< 0.001**	0.56	**6.17**	**0.014**	0.61

F-Fishera-Snedecor test value; p- probability, Note - statistically significant differences are marked in bold

As a result of contrast analysis eight significant results (α = 0.05) were found in twelve test performed (Table 4). Probability of obtaining such number of significant differences among twelve multiple comparisons P_B_ (A) = 1,57E-08 is very low. Therefore, it was assumed that the observed differences reflected actual biological effects.

2D: 4D R digit ratio showed no dimorphic differences in the migraine group (*p* = 0.500), whereas in the control group differences were statistically significant (*p* < 0.001) and their direction were as expected (females had higher mean of 2D:4D R than males).

**Fig. 1:**
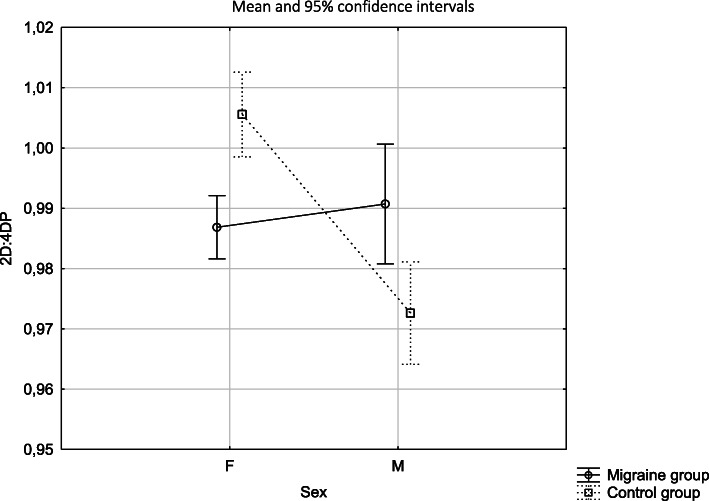
2D:4D of the right hand depending on sex and the study group

Comparison 2D:4D R in both study groups showed that females with migraine had lower 2D:4D R than females in the control group (*p <* 0.001), whereas males with migraine had higher 2D:4D R than males in the control group (*p* = 0.007). Consequently the risk of migraine was increased in females with lower 2D:4D R (OR 2.98, 95% CI 1.54–5.76) and among males the risk of migraine was increased when 2D:4D R was higher (OR 1.48, 95% CI 0.49–4.46) (Tables 3, 4; Fig. 1).

**Fig. 2:**
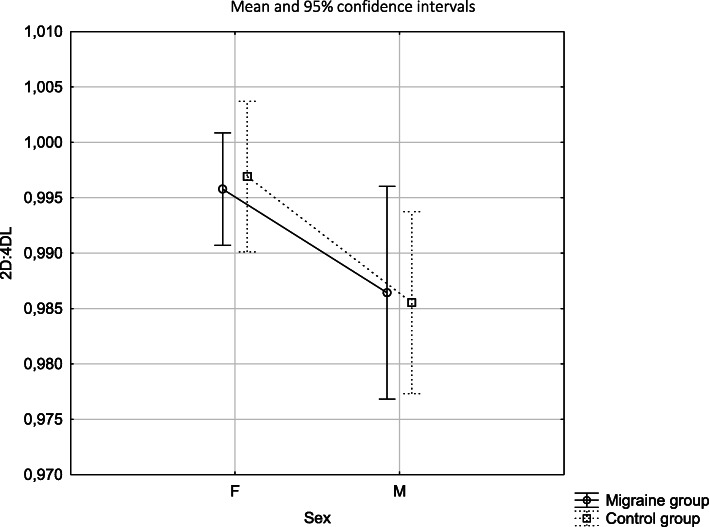
2D:4D of the left hand depending on sex and the study group

2D: 4D L digit ratio showed no dimorphic differences in the migraine group (*p* = 0.091) and it was differentiated between females and males in the control group (*p* = 0.036), presenting as expected higher values for females and lower values for males. At the same time 2D: 4D L turned out to be similar in females in the both study groups (*p* = 0.793) and in males in the both study groups (*p* = 0.889) (Tables 3, 4, Fig. 2).

**Fig. 3:**
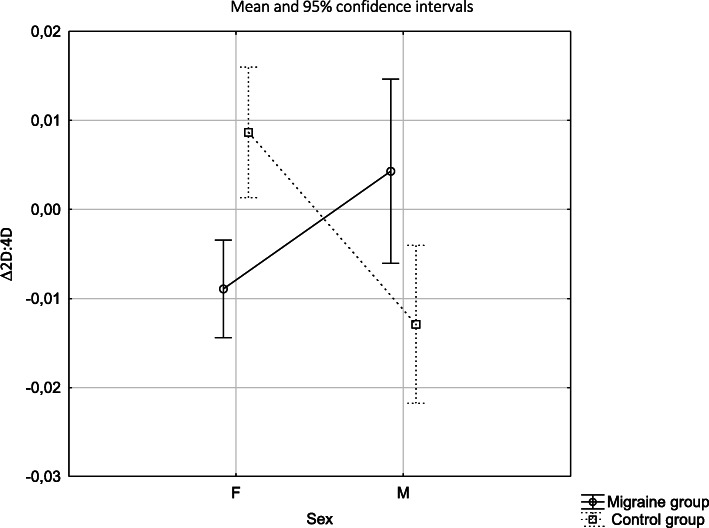
Difference between 2D:4D R and 2D:4D L depending on sex and the study group

Δ2D:4D showed sexual dimorphism in both study groups, but in females with migraine it was characterized by lower values than in males with migraine (*p* = 0.027), whereas in the control group the opposite direction of the differences was observed (*p* < 0.001). Females with migraine had lower Δ2D:4D than females in the control group (*p <* 0.001). Males with migraine had higher Δ2D:4D than males in the control group (*p* = 0.014) (Tables 3, 4; Fig.3).

## Discussion

In the prenatal period a foetus is more sensitive to variety of factors, including hormonal disturbances [47–49]. Intrauterine hormonal environment is probably associated with a number of diseases in adulthood, and migraine can be considered as one of them. Migraine is a common health problem and can be determined already in early ontogenesis, affecting about 10% of children and adolescents [4, 8]. Manning and Bundred (2000) have drawn attention to a role of prenatal hormonal environment in developing several diseases, and suggested 2D:4D digit ratio as a potential predictor of them [50]. 2D:4D ratios are stable during postnatal ontogenesis and are established already at the prenatal stage of the development. Lower values of 2D:4D digit ratio are linked with high testosterone levels during pregnancy. Testosterone has an impact on the central nervous system by slowing down the growth of the left hemisphere (and promotes the growth of the right hemisphere simultaneously). Geschwind and Galaburda suggested that there is an association between prenatal testosterone migraine and left-handedness [34]. Masculinized 2D:4D has been associated in women with breast cancer [30], lung cancer [33], and carpal tunnel syndrome [51] and prostate cancer in men [52]. Feminized 2D:4D has been linked to heart disease in men [53] and greater pain resistance in women [54].

The influence of the sexual dimorphism on the prevalence of migraine is probably multifactorial, but many researchers suggest an important role of sex hormones [55, 56]. However, the role of sex steroids at the prenatal stage is not fully understood.

The aforementioned data on prenatal hormonal environmental conditions as a risk factor of migraine published by Xie et al. showed that prenatal testosterone dominance can lead to increased prevalence of migraine in females [22]. In the Chinese population women with masculinized digit ratio 2D:4D R & L tended to suffer more from migraine and tension type headache (TTH), but there was no such relationship in males [22]. The results of our study suggest that prenatal sex steroids exposure has influence on the risk of migraine both in women and in men. Our results in the group of female migraineurs were similar to the data of Xie et al. and thus masculinized digit ratios were associated with migraine prevalence (OR = 2.98), but significant relationship was noticed only in the right hand. It should be emphasized however, that the right hand is regarded as a better indicator of androgenisation than the left one [25]. What is new what we have shown is that prenatal oestrogen exposure probably can also increase the risk of migraine, and that such relationship is sex-dependent and observed only in male migraineurs in the right hand (OR = 1.48).

The results of our study showed that both 2D:4D digit ratio and Δ2D:4D were significantly associated with migraine. The Δ2D:4D was first considered to be an additive correlate of prenatal oestrogen-testosterone balance by Manning et al. in 2000 [28]. Low Δ2D:4D is associated with prenatal testosterone dominance. Its value tends to be negative in men (lower 2D:4D R than 2D:4D L) and positive in women (higher 2D:4D R than 2D:4D L) [27]. The results of this study showed such sex differences pattern in the control group (male Δ2D:4D < female Δ2D:4D). The opposite result was found in the migraine group (male Δ2D:4D > female Δ2D:4D). Our data provide the new valuable link between high prenatal oestrogen exposure in men and migraine.

Study by Manning & Peters (2009) showed that low Δ2D:4D is connected with left-handedness [29]. Left hand preference is also associated with migraine [34]. In 1982 Geshwind and Behan reported higher frequency of left-handed individuals in migraineurs [57]. The latest study published in 2021 showed that handedness is also possibly connected with pain localization during migraine attacks. Over 60% attacks characterized by pain on the right side were related to the right-handedners (62.8%) and attacks characterized by pain on the left side were related to the left-handedners (63.5%) [58].

The fact of a relationship between migraine and sex hormones levels has been recognized by many epidemiological studies. Especially in women, oestrogen fluctuations are identified as a risk factor of migraine and migraine attacks [56]. Pourabolghasem et al. suggested that there is no evidence that higher levels of male sex hormones (mainly testosterone) in female migraineurs with polycystic ovary syndrome (PCOS) intensify migraine attacks [59]. Glintborg et al. reported that migraine was more prevalent in PCOS females [60]. Sir-Peterman et al. showed that among pregnant women with PCOS peripheral serum androgen concentrations were significantly elevated and constituted a potential source of excessive androgen for a foetus [61]. PCOS belongs to the most common endocrine disorders affecting women of childbearing potential, and women suffering from it who wish to become pregnant or already pregnant women should be monitored with special care.

Previously it has been shown that oestrogen can influence initiating migraine attack in women [62]. Recently it has turned out that the female sex hormones also affect male migraineurs when oestrogen is elevated - van Oosterhout reported that men with migraine (with at least 3 episodes per month) had increased levels of the oestrogen (E2) and androgen deficiency at the same time (*N* = 17) [63]. Shields et al. reported in their pilot study that men with chronic migraine (*N* = 14) also had decreased testosterone levels [64]. Our study indirectly reveals that oestrogen-testosterone balance in men with migraine is also associated with oestrogen dominance, but already at the prenatal stage of development. The role of oestrogen and testosterone in modulating migraine deserves further investigations whether there is a link between prenatal exposure and postnatal levels or not.

There is a need of indicators that would be helpful in assessing lifelong risks of prenatal disturbances that can increase migraine prevalence. Hormonal environment in utero seems to play significant but unexplored role in developing migraine. Prenatal testosterone and estrogen levels estimated indirectly by 2D:4D digit ratio can be considered as one of such indicators for both sexes.

## Conclusions

Although the relationship between migraine and sex hormones has already been demonstrated many times, pathophysiology of this relationship has not yet been fully explained. The role of sex steroids at prenatal stage for development of migraine is not understood. Migraine can be “sex-steroid-programmed” in foetal life differently in men and women. Increased prenatal testosterone levels in women significantly correlated with the prevalence of migraine. In men, the opposite correlation was observed: males with presumably decreased testosterone levels in prenatal life are supposed to suffer more probably from migraine in adulthood. This research should be continued by expanding study groups and analysing correlations in different populations.

### Limitations

Study was limited by a small sample of men who suffer from migraine. Patients were from quite homogenous population in Poland.

## Data Availability

The datasets used in the current study available from the corresponding author on request.
